# Prognostic and immune predictive roles of a novel tricarboxylic acid cycle-based model in hepatocellular carcinoma

**DOI:** 10.1038/s41598-024-52632-0

**Published:** 2024-01-28

**Authors:** Yifan Zeng, Tao Yu, Shuwen Jiang, Jinzhi Wang, Lin Chen, Zhuoqi Lou, Liya Pan, Yongtao Zhang, Bing Ruan

**Affiliations:** State Key Laboratory for Diagnosis and Treatment of Infectious Diseases, National Clinical Research Center for Infectious Diseases, National Medical Center for Infectious Diseases, Collaborative Innovation Center for Diagnosis and Treatment of Infectious Diseases, The First Affiliated Hospital, Zhejiang University School of Medicine, 79 Qingchun Rd., Hangzhou City, 310003 China

**Keywords:** Computational biology and bioinformatics, Biomarkers, Oncology

## Abstract

Hepatocellular carcinoma (HCC) is the most prevalent type of liver cancer. Since the tricarboxylic acid cycle is widely involved in tumor metabolic reprogramming and cuproptosis, investigating related genes may help to identify prognostic signature of patients with HCC. Data on patients with HCC were sourced from public datasets, and were divided into train, test, and single-cell cohorts. A variety of machine learning algorithms were used to identify different molecular subtypes and determine the prognostic risk model. Our findings revealed that the risk score (TRscore), based on the genes OGDHL, CFHR4, and SPP1, showed excellent predictive performance in different datasets. Pathways related to cell cycle and immune inflammation were enriched in the high-risk group, whereas metabolism-related pathways were significantly enriched in the low-risk group. The high-risk group was associated with a greater number of mutations of detrimental biological behavior and higher levels of immune infiltration, immune checkpoint expression, and anti-cancer immunotherapy response. Low-risk patients demonstrated greater sensitivity to erlotinib and phenformin. SPP1 was mainly involved in the interaction among tumor-associated macrophages, T cells, and malignant cells via SPP1–CD44 and SPP1–(ITGA5 + ITGB1) ligand-receptor pairs. In summary, our study established a prognostic model, which may contribute to individualized treatment and clinical management of patients with HCC.

## Introduction

Primary liver cancer is the fifth most frequently occurring cancer globally^1,2^. Hepatocellular carcinoma (HCC) is the most prevalent type of liver cancer, making up more than three-quarters of cases worldwide^3^. Currently, surgical treatment remains the treatment of choice option for liver cancer; however, a significant number of patients are deemed ineligible for surgery after evaluation^4,5^. To increase the overall survival rates of patients with liver cancer, several systemic treatments are being developed, including local ablation, arterial embolization, radiotherapy, targeted drugs, immune-based therapies, and combination chemotherapy. In the highly successful IMbrave-150 trial enrolling patients with unresectable liver cancer, treatment with combination chemotherapy of atezolizumab plus bevacizumab achieved an objective response rate of 30%^6^. Irrespective, the overall prognosis for liver cancer remains suboptimal owing to a lack of definitive biomarkers or systematic staging systems to accurately evaluate the disease. Therefore, further exploration into additional biomarkers is necessary to improve prognostic assessment and analysis.

Since 2000s, metabolic reprogramming has been recognized as a common characteristic of several tumors^7^. Evidence suggests that tumor cells adapt their metabolic programming to fulfill the energy and biosynthetic requirements for growth and proliferation. Reportedly, tumor cells break down nutrients absorbed from the tumor microenvironment (TME) into various intermediates for further biosynthesis as well as rely heavily on the glycolysis pathway for glucose metabolism. In 1956, Warburg proposed the phenomenon of “aerobic glycolysis” in tumor cells, which has since been extensively investigated from different perspectives. One study has found that the mitochondrial tricarboxylic acid (TCA) cycle pathway is inhibited in tumor cells, which significantly reduces the entry of pyruvate into this cycle relative to glucose intake. This inhibition may be associated with excessive activation of pyruvate kinase M2^8^. In addition, studies have reported the presence of abnormalities in TCA cycle enzymes in tumors. A retrospective study enrolling patients with HCC who underwent transcatheter arterial chemoembolization identified isocitrate dehydrogenase (IDH) as a potential prognostic marker^9^. Reportedly, numerous reductive biosynthetic reactions occurring during tumor proliferation necessitate the involvement of reducing substances such as NADPH. In tumor cells, NADPH is primarily derived from the controlled oxidation of carbon substrates^10^. Moreover, a previous study investigating the role of an NADPH producer, the malic enzyme family, found that adaptive and constitutive activation of malic enzymes could provide multiple layers of protection against oxidative stress in HCC cells^11^. This highlights that metabolic reprogramming is an central component in the development of HCC, suggesting that the TCA cycle is closely associated with the growth and metastasis of tumors^12^. However, the relationship between related genes and the prognosis of patients with HCC has not been extensively explored.

The TME of HCC is composed of complex cellular and non-cellular components that play a crucial role in immune evasion, metastasis, and drug resistance. An imbalance in the microenvironment caused by divergent factors is one of the key mechanisms of tumorigenesis^13^. A previous study has revealed the protective role of fumarate hydratase (FH), a TCA cycle enzyme, in regulating macrophage cytokine and interferon responses^14^. Moreover, metabolic reprogramming, including TCA cycle remodeling, may alter the metabolism and effector functions of immunocytes such as macrophages^15^. Therefore, a comprehensive analysis of related genes will help predict the immune microenvironment of HCC and assess the effect of immunotherapy.

Based on previous findings, the present study attempted to construct a novel prognostic model of HCC based on public transcriptomics data. Furthermore, the model was evaluated and validated based on the immune microenvironment, immunotherapy response, mutation characteristics, clinicopathological features, drug sensitivity, and cell communication. Although previous studies have preliminarily explored the effects of cuproptosis and the TCA cycle on the prognosis of patients with HCC, these studies primarily focused on cuproptosis, an emerging form of cell death^16–18^. In contrast, the present study will attempt to gain a broader perspective of genes and microenvironment based on the TCA cycle, which will help provide novel strategies for making treatment decisions.

## Results

### Molecular subtypes derived from TCA cycle-related genes

**Figure 1:**
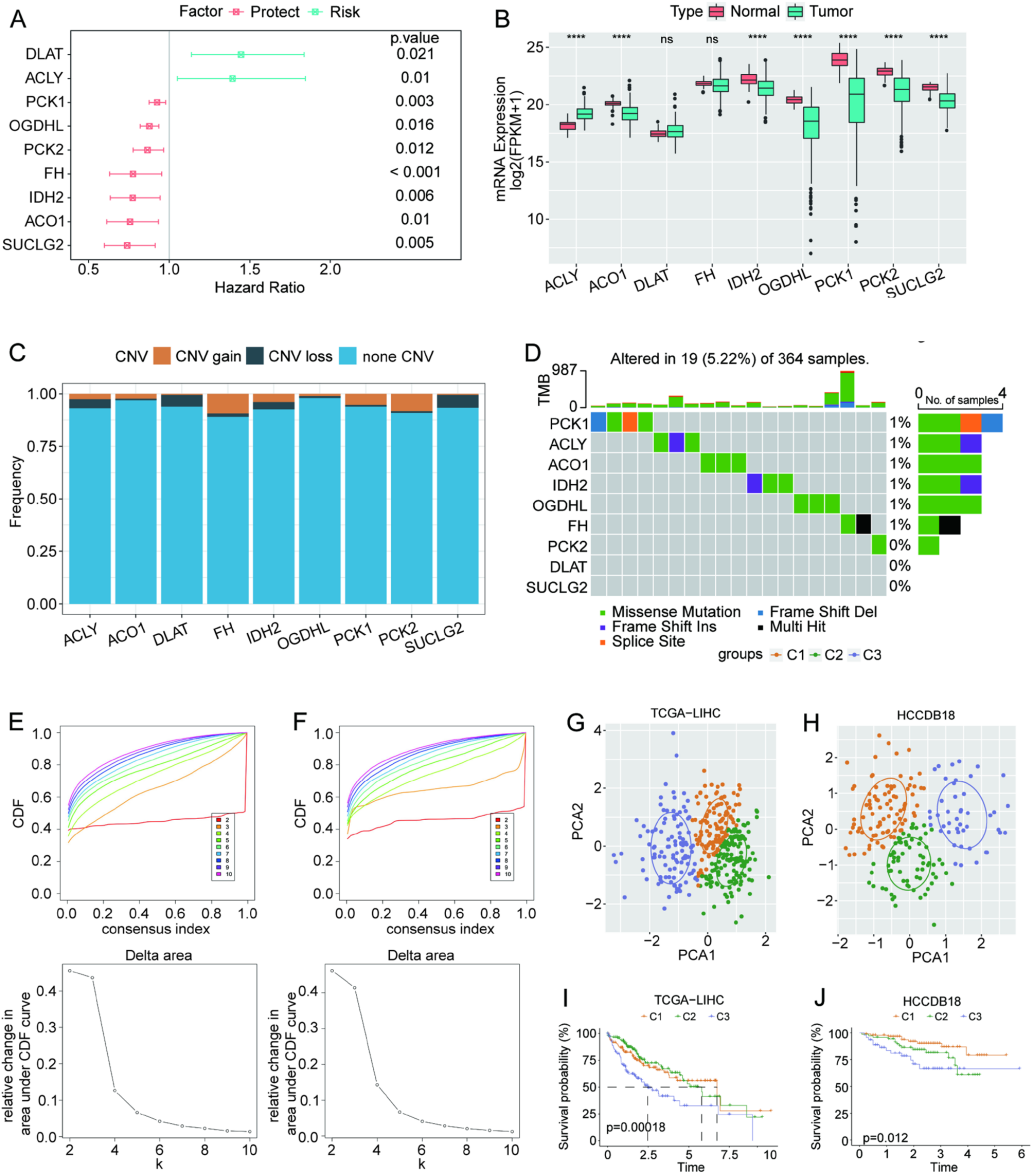
Gene expression and consensus clustering of patients with HCC based on TCA cycle-related genes. (**A**) Univariate forest plot of TCA cycle-related genes with significant prognosis. (**B**) Differential gene expression of potential prognostic genes in HCC tissues and adjacent non-tumor tissues. (**C**) The frequency of CNV mutations. (**D**) Analysis of somatic mutations. (**E**,**F**) The cumulative distribution function (CDF) curve and delta area curve of consensus clustering. (**G**,**H**) Principal component analysis in the (**G**) TCGA-Liver Hepatocellular Carcinoma (TCGA-LIHC) and (**H**) HCCDB18 cohorts. (**I**,**J**) Kaplan–Meier survival analysis.

To investigate the involvement of the TCA cycle pathway in HCC, we analyzed the expression of related genes using the The Cancer Genome Atlas Liver Hepatocellular Carcinoma (TCGA-LIHC) dataset. Univariate COX analysis was performed on 31 genes related to the TCA cycle. The results showed that nine genes were significantly associated with the prognosis of HCC: *DLAT* and *ACLY* were risk factors, whereas *PCK1, OGDHL, PCK2, FH, IDH2, ACO1,* and *SUCLG2* were protective factors (Fig. 1A). Among them, as typical representatives, ATP citrate lyase (*ACLY*) is highly expressed in the adipose tissue and liver, and oxoglutarate dehydrogenase-like (*OGDHL*) exhibits a tumor suppressor effect in HCC, which is consistent with the results of our preliminary exploration^19^. We demonstrated that seven genes were differentially expressed between HCC and adjacent tissues, with *ACO1, IDH2, OGDHL, PCK1, PCK2,* and *SUCLG2* being highly expressed in normal tissues (Fig. 1B). An analysis of the data on gene stable nuclear variant (SNV) and copy number variation (CNV) mutations in HCC revealed that the gene copy numbers were not significantly amplified or reduced (Fig. 1C). Moreover, the nine prognostic genes demonstrated a very low mutation frequency. The waterfall diagram demonstrated that 19 of the 364 samples with HCC (5.22%) displayed mutations in TCA cycle-related genes (Fig. 1D).

Next, we analyzed the samples in the TCGA-LIHC dataset via consistent clustering based on the nine prognostic genes identified above. the cumulative distribution function (CDF) delta area curve revealed that setting the cluster at 3 generates a more stable clustering result (Fig. 1E–F). Similar results were obtained in the validation dataset HCCDB18. Therefore, we acquired three molecular subtypes (C1, C2, and C3), and principal component analysis (PCA) was applied to verify the rationality of the classification. As expected, the three subtypes demonstrated extremely clear boundaries in the TCGA-LIHC and HCCDB18 datasets (Fig. 1G–H). Moreover, survival analysis demonstrated that the C1 subtype had the best prognosis and C3 had the worst prognosis (Fig. 1I–J).

### Immune signatures among molecular subtypes and identification of differentially expressed genes (DEGs)

**Figure 2:**
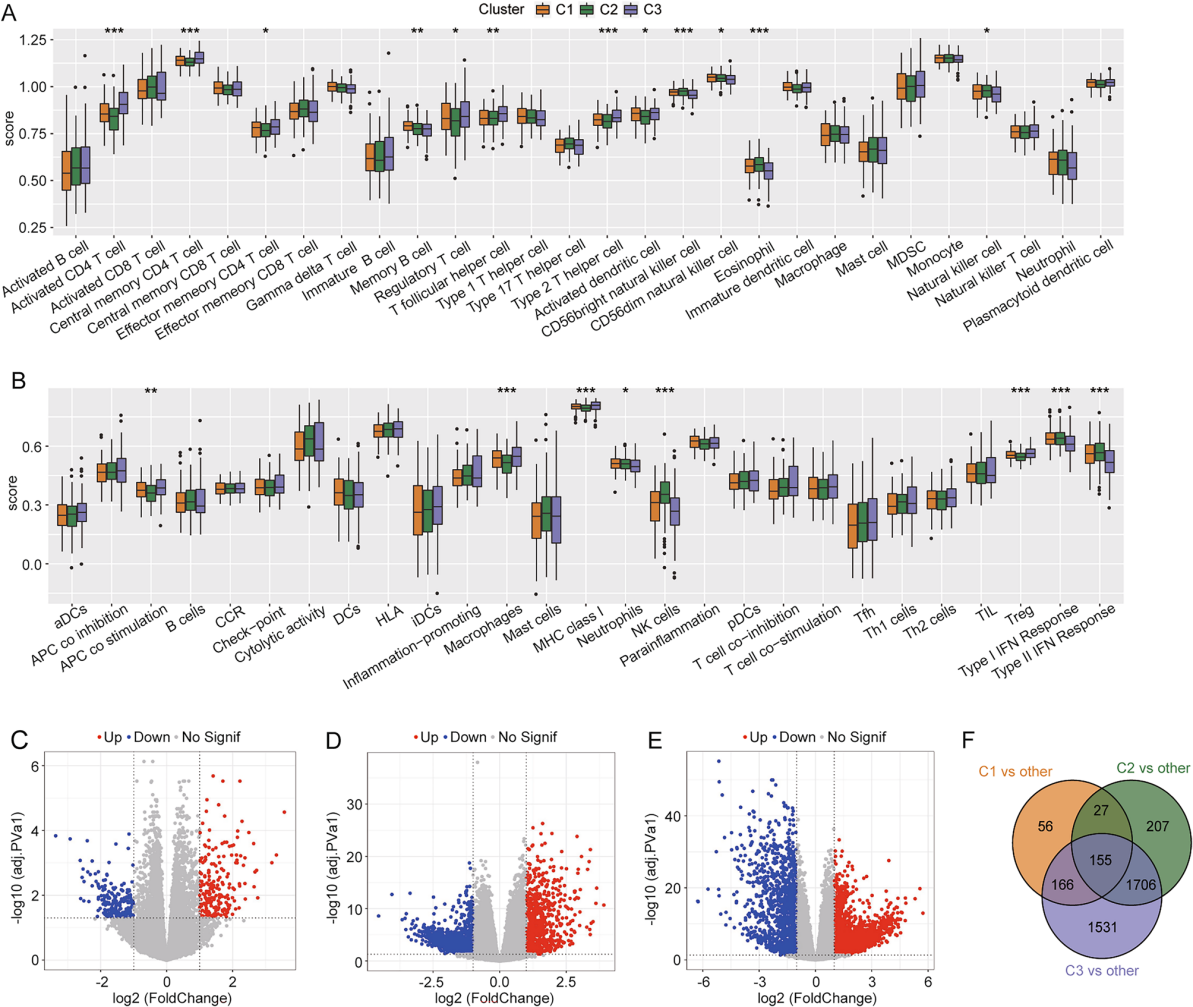
Comparison of immune infiltration among three molecular subtypes and identification of differentially expressed genes (DEGs). (**A**) Comparison of 28 immune cells evaluated using ssGSEA. (**B**) Comparison of 27 immune components. (**C**–**E**) Volcanic plot of DEGs between (C) C1 and other clusters; (**D**) C2 and other clusters; and (**E**) C3 and other clusters. (**F**) Venn diagram of DEGs.

In addition to tumor cells, the TME comprises several stromal cells, such as fibroblasts and immunocytes. Particularly, immunocytes play important contradictory anti-tumor and tumor protective roles. To explore the activity of immunocytes and their functions or pathways in tumor samples, we performed single-sample gene set enrichment analysis (ssGSEA). The box plot demonstrated differences in immune infiltration between C1, C2, and C3 subtypes. The C3 subtype was associated with a higher activity of T cells and macrophages but a lower activity of natural killer cells and neutrophils. Moreover, this subtype was associated with higher enrichment levels of “MHC class I” and “APC co-stimulation” but lower enrichment levels of certain functions, including “type I IFN response” and “type II IFN response” (Fig. 2A–B). Tumor Immune Estimation Resource (TIMER) analysis also illustrated significant differences in immune-infiltrating cells among subtypes (Fig. S1).

In total, 155 DEGs were found to be common among the C1, C2, and C3 subtypes, which provided a basis for further construction of the prognostic model (Fig. 2C–F).

### Establishment and verification of risk model

The risk score model was constructed using the TCGA-LIHC cohort, and the samples were categorized into train (n = 182) and test cohorts (n = 183). The chi-square test revealed no significant difference in the clinical feature between the two groups (*p* > 0.05, Table 1), indicating that the grouping was random and reasonable.

**Table 1:** Clinical characteristics of patients with HCC in train and test group.

Characteristics	Train (N = 182)	Test (N = 183)	Total (N = 365)	pvalue	FDR
Age					
Mean ± SD	58.71 ± 14.01	60.57 ± 12.65	59.65 ± 13.36		
Median[min–max]	61.00 [16.00,90.00]	61.00 [17.00,85.00]	61.00 [16.00,90.00]		
Gender				1	1
Female	59 (16.16%)	60 (16.44%)	119 (32.60%)		
Male	123 (33.70%)	123 (33.70%)	246 (67.40%)		
AJCC stage				0.69	1
I	90 (24.66%)	80 (21.92%)	170 (46.58%)		
II	41 (11.23%)	43 (11.78%)	84 (23.01%)		
III	36 (9.86%)	47 (12.88%)	83 (22.74%)		
IV	2 (0.55%)	2 (0.55%)	4 (1.10%)		
NA	13 (3.56%)	11 (3.01%)	24 (6.58%)		
Grade				0.97	1
G1	28 (7.67%)	27 (7.40%)	55 (15.07%)		
G2	89 (24.38%)	86 (23.56%)	175 (47.95%)		
G3	56 (15.34%)	62 (16.99%)	118 (32.33%)		
G4	6 (1.64%)	6 (1.64%)	12 (3.29%)		
NA	3 (0.82%)	2 (0.55%)	5 (1.37%)		

*FDR* false discovery rate, *SD* standard deviation, *NA* not available.

**Figure 3:**
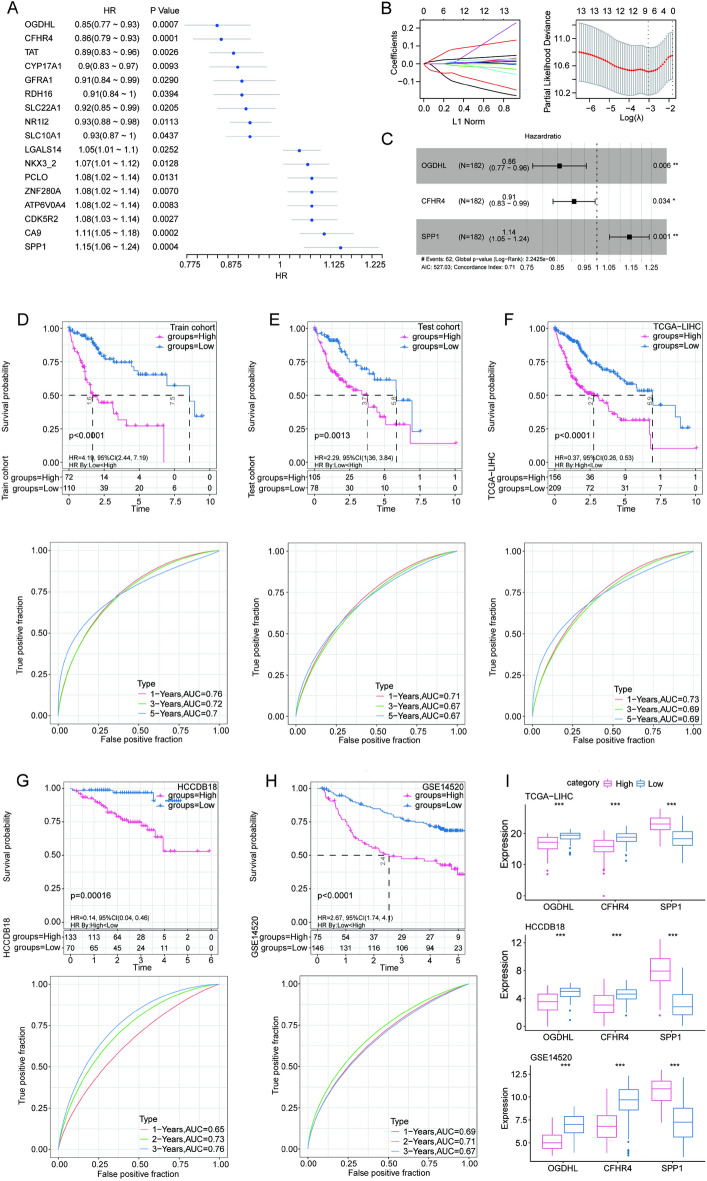
Establishment and validation of risk model. (**A**) Univariate forest plot of DEGs in the training cohort. (**B**) Lasso–Cox regression further narrowed the gene range. (**C**) Risk coefficients based on multivariate Cox regression analysis. (**D**–**H**) The Kaplan–Meier survival curves and receiver operating characteristic curves in the (**D**) train, (**E**) test, (**F**) TCGA–LIHC, (**G**) HCCDB18, and (**H**) GSE14520 cohorts. (**I**) The expression of three model genes in different datasets.

To further determine the key gene sets, 17 prognosis-related genes were identified from the DEGs determined from the TCGA–LIHC training cohort, using univariate Cox analysis (*p* < 0.05) (Fig. 3A). After a preliminary screening of genes by LASSO regression, stepwise multivariate regression analysis further identified *OGDHL, CFHR4,* and *SPP1* as prognostically significant signatures (Fig. 3B–C) and were used to build the TCA cycle-based risk model. Furthermore, an innovative risk scoring system, called TRscore, was used to quantify the prognostic risk of patients with HCC. Then, the risk score for each sample was calculated based on the following formula:

$$TRscore \, = \, - 0.15384795 \, \times OGDHL + \, \left( { - 0.09394254} \right) \, \times CFHR4 + \, 0.13353913 \, \times SPP1$$

For each patient in the train, test, TCGA–LIHC, and external validation datasets (HCCDB18 and GSE14520), a TRscore was calculated. According to the optimal cut-off value, patients with HCC were further divided into high- and low-risk groups. Next, we performed time-dependent receiver operating characteristic (ROC) curve analysis in these cohorts to evaluate the predictive accuracy of the risk model. As shown in the Fig. 3D–E, the model had a higher area under the curve, and the high-risk group had a worse prognosis. In almost all datasets, the model demonstrated high classification efficiency of 1-, 3-, and 5-year prognosis (Fig. 3F–H). Finally, the assessment of expression of the three model genes in different datasets revealed that *SPP1* was highly expressed in the high-risk group, and *OGDHL* and *CFHR4* were highly expressed in the low-risk group (Fig. 3I); this finding was consistent with the results of the hazard ratio. In summary, the TRscore can accurately predict the prognosis of patients with HCC.

**Figure 4:**
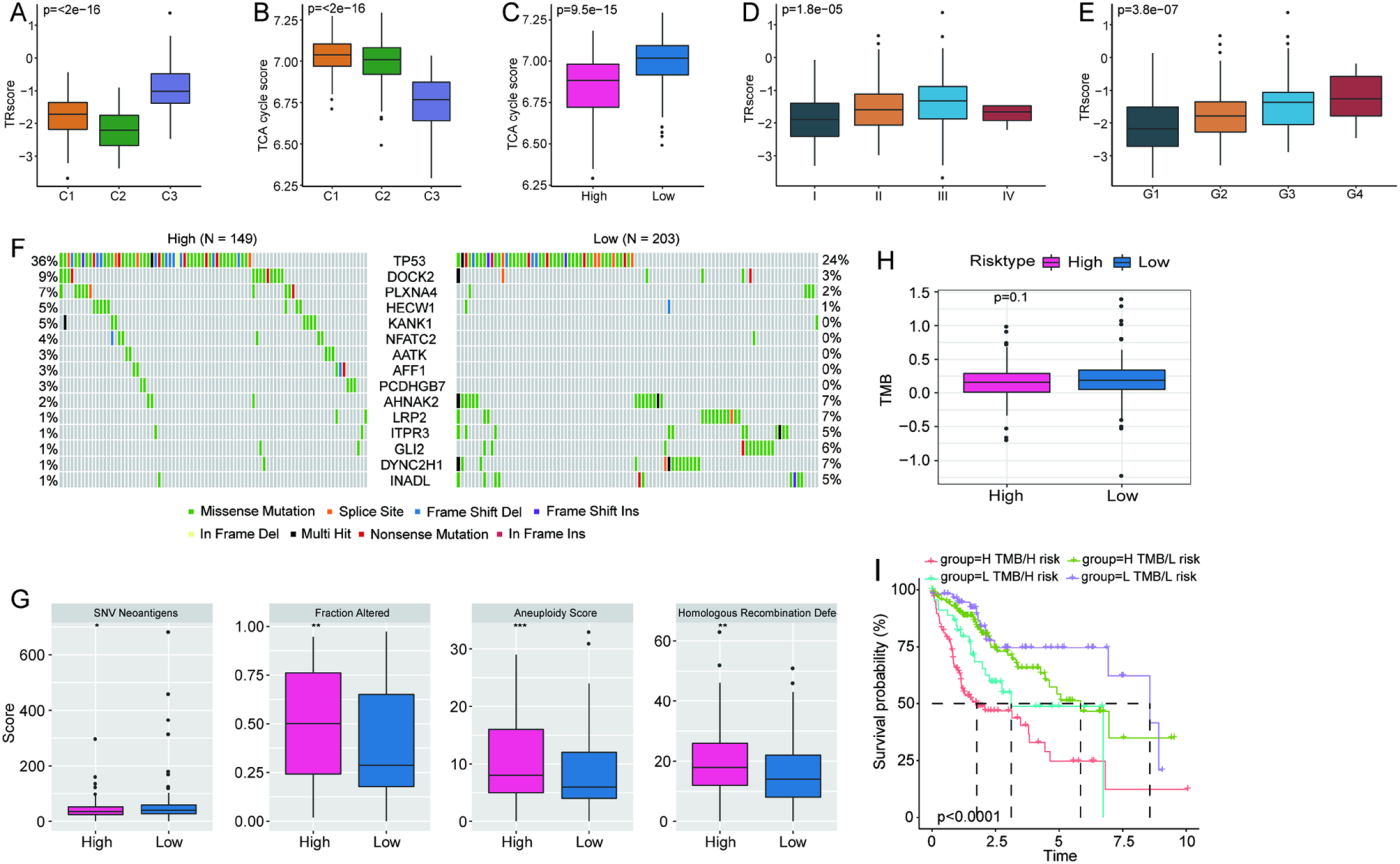
Clinicopathological characteristics and mutation characteristics. (**A**,**B**) Distribution of (**A**) TRscore and (**B**) TCA cycle-related gene enrichment score in different molecular subtypes based on the TCGA–LIHC cohort. (**C**) The distribution of TCA cycle-related gene enrichment score according to different risk statuses. (**D**,**E**) The distribution of TRscore in different clinicopathological characteristics. (**F**) Characteristics of somatic mutations in the high- and low-risk groups. (**G**) Differences in mutation characteristics between high- and low-risk groups. (**H**) The distribution of TMB according to risk. (**I**) The Kaplan–Meier survival curves of risk groups combined with TMB.

The new risk model based on the TCA cycle can determine the relationship between the prognosis of patients with HCC and marker genes. Further, we compared the differences in TRscore and TCA cycle-related gene enrichment score (hereinafter referred to as TCA cycle score) among the three subtypes in the TCGA–LIHC dataset. The results showed that for the C3 subtype, the TRscore was higher with the worst prognosis (Fig. 4A), whereas the TCA cycle score was the opposite (Fig. 4B). In addition, comparing the difference in TCA cycle scores revealed that the score in the low-risk group was significantly higher than that in high-risk group (Fig. 4C).

To further elucidate the relevance of the risk model and clinicopathological features in HCC, we compared the distribution of TRscore among different clinical parameter groups defined by the American Joint Committee on Cancer (AJCC) stage and Grade for HCC^20^. We noted significant differences in the TRscores of different pathological features. Moreover, the TRscore generally showed an upward trend with a higher staging and grading (Fig. 4D–E).

### Comparison of mutation characteristics and immunotherapy response

To evaluate the role of somatic mutations in cancer progression, we described the gene mutation landscapes between the high- (n = 149) and the low-risk groups (n = 203) in the TCGA cohort. The mutation characteristics of the first 15 genes are shown in the waterfall diagram (Fig. 4F). Mutations associated with detrimental biological behavior were more abundant in the high-risk group than in the low-risk group, such as *TP53* (36% vs. 24%), a well-known anti-oncogene that is one of the most frequently mutated genes in HCC specimens, and *DOCK2* (9% vs. 3%), a mediator of cytokinesis^21^. According to the genomic characteristics of HCC mutations obtained from the previous study, we noted significant differences in the aspects of SNV neoantigens, fraction altered, aneuploidy score, and homologous recombination defects between the two groups (Fig. 4G)^22^.

Tumor mutational burden (TMB) is an important indicator of tumor progression and an emerging biomarker for evaluating the response to immune checkpoint inhibitors (ICIs). We found no significant difference in the TMB between the low- and the high-risk groups (Fig. 4H). However, after categorizing HCC samples into four subgroups based on high/low TMB and TRscore, we found that the L-TMB + L-risk group had the best prognosis, whereas the H-TMB + H-risk group had the worst prognosis (Fig. 4I).

**Figure 5:**
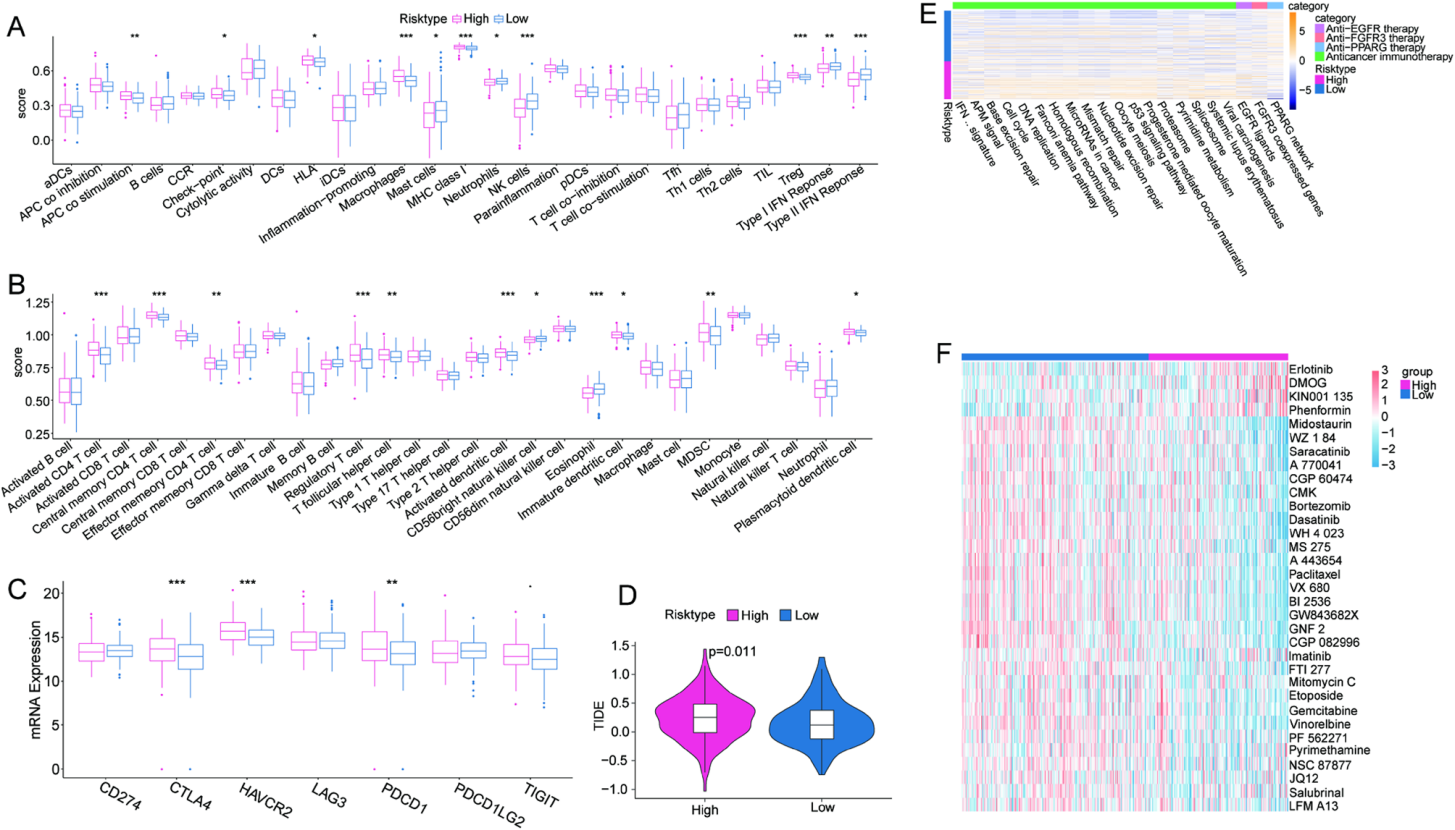
Prediction of immune infiltration and immunotherapy response using TRscore in the TCGA-LIHC dataset. (**A**) Comparison of 27 immune components assessed using ssGSEA. (**B**) Comparison of 28 immune cells. (**C**) Gene expression of common immune checkpoints. (**D**) TIDE analysis under different risk statuses. (**E**) Prediction score of the immunotherapy. (**F**) The sensitivity of patients with HCC to traditional chemotherapeutic drugs.

In recent years, immunotherapy combined with ICIs has demonstrated surprising prognostic efficacy in clinical treatment of cancers. Accordingly, in the present study, immune infiltration analysis showed that the immune score of the high-risk group was higher on the whole than that of the low-risk group (Fig. 5A–B). The immune status of the TME provides a reference for tumor immunotherapy. Analyzing the expression of several common immune checkpoint genes revealed that the high-TRscore group had higher levels of immune checkpoint molecules such as CTLA4, HAVCR2, PDCD1, and TIGIT (Fig. 5C). Moreover, the high-risk HCC group was more likely to demonstrate inhibition of T-cell function and escape from anti-tumor immunity, which additionally suggested that such high-risk patients may respond more actively to immune checkpoint therapy. Besides, the tumor immune dysfunction and exclusion (TIDE) algorithm predicts responses to immunotherapy, with higher scores indicating a higher possibility of immune escape, thus indicating a lower benefit of immunotherapy. Patients with low TRscores in the TCGA cohort tended to have lower TIDE scores, suggesting that low-risk populations were more likely to benefit from immunotherapy (Fig. 5D). Next, the therapeutic predicted pathways signatures were obtained from the previous study and scored using the ssGSEA method revealed that the activity of anticancer immunotherapy was higher in the high-risk group than in the low-risk group (Fig. 5E)^23^.

In summary, the combination of TRscore and TMB can be used to stratify HCC subgroups with the worst prognosis. In addition, the model can help indicate whether patients with HCC can benefit from immunotherapy and ICI therapy.

### Evaluation of drug sensitivity and analysis of biological pathways

An evaluation of the sensitivity to traditional chemotherapeutic drugs revealed that the high-risk group was sensitive to 29 of the 33 drugs, including midostaurin, saracatinib, bortezomib, and dasatinib, whereas the low-risk group was sensitive to only four drugs. The identification of candidates with better antitumor response will help guide personalized drug therapy for patients with HCC in clinical practice (Fig. 5F).

**Figure 6:**
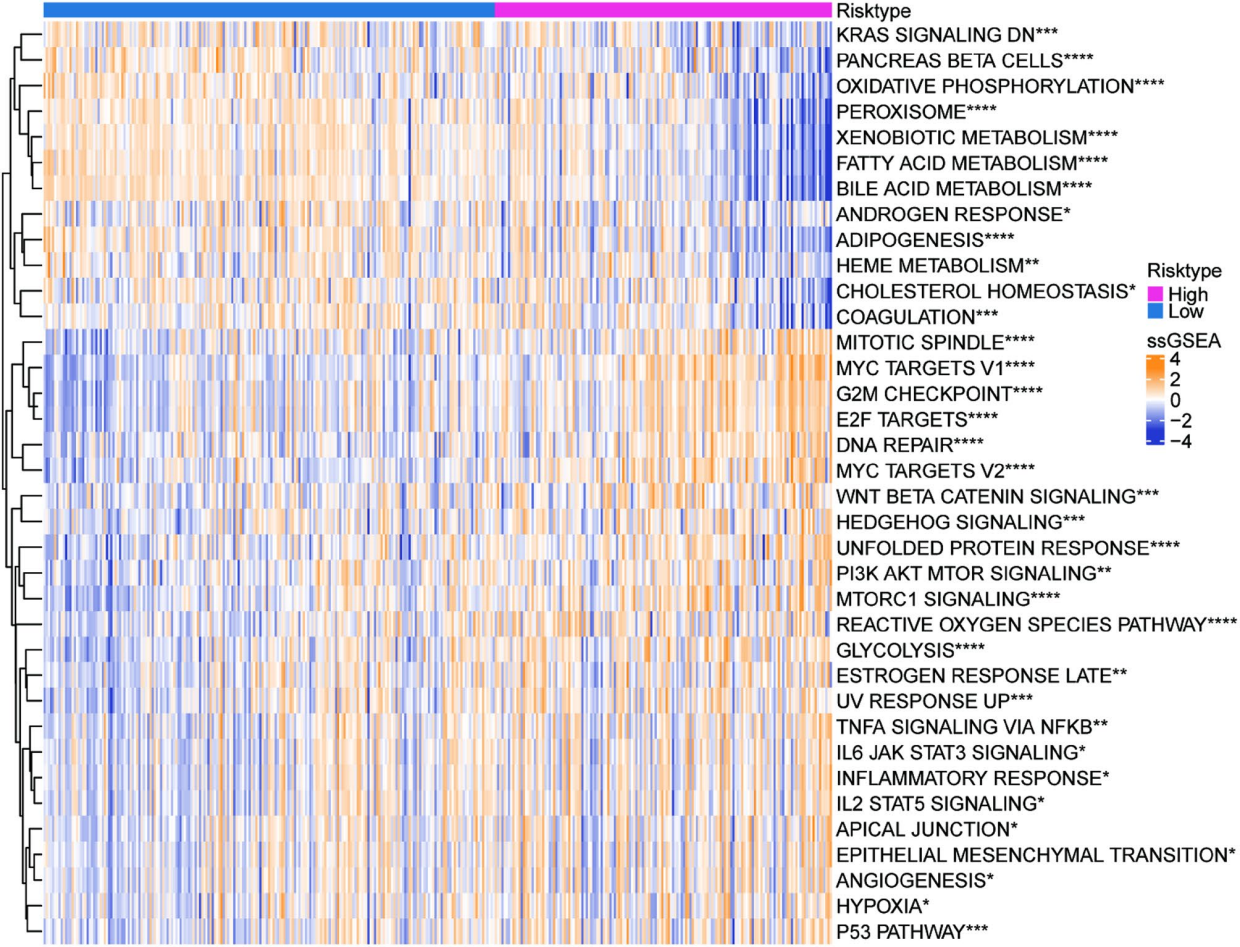
Biological functions enriched in different risk subgroups in the TCGA-LIHC dataset.

To further explore the differences in gene transcription levels between samples in high- and low-risk groups, gene enrichment analysis was performed on the TCGA dataset using the ssGSEA method. The heat map displayed 36 pathways with significant differences (Fig. 6). Cell cycle-, immune inflammation-, and tumor development-related pathways were more active in the high-risk group, whereas metabolism-related pathways, including xenobiotic metabolism, fatty acid metabolism, bile acid metabolism, and adipogenesis, were significantly enriched in the low-risk group. Similar results were obtained in the validation datasets HCCDB18 (Fig. S2A) and GSE14520 (Fig. S2B).

### Pan-cancer analysis of the TRscore risk model

**Figure 7:**
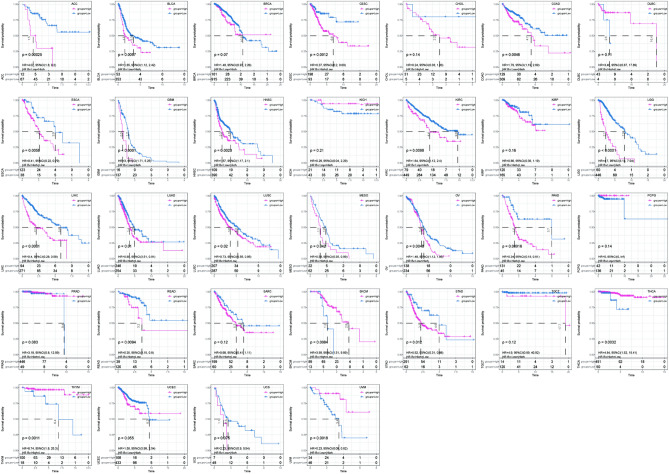
Performance efficiency of the TRscore in predicting patient outcome across cancer types.

Based on the pan-cancer multi-omics data obtained from TCGA, we further analyzed the efficiency of TRscore in predicting the prognosis of patients. Using the model, the TRscore of each cancer type was calculated and the best cutoff value was determined to analyze the survival curve. The results demonstrated that the TRscore showed a wide range of universality in 32 cancer types. TRscore demonstrated significant differences in the prognosis of different risk groups enrolling patients with 21 cancer types, including adenoid cystic carcinoma, low-grade glioma, cervical squamous cell carcinoma, and pancreatic adenocarcinoma. In most cancer types, higher TRscores were associated with poorer outcomes of patients (Fig. 7). At the same time, the ROC curves of each cancer type were shown in Fig. S3.

### TRscore serves as a valuable prognostic factor for patients with HCC

**Figure 8:**
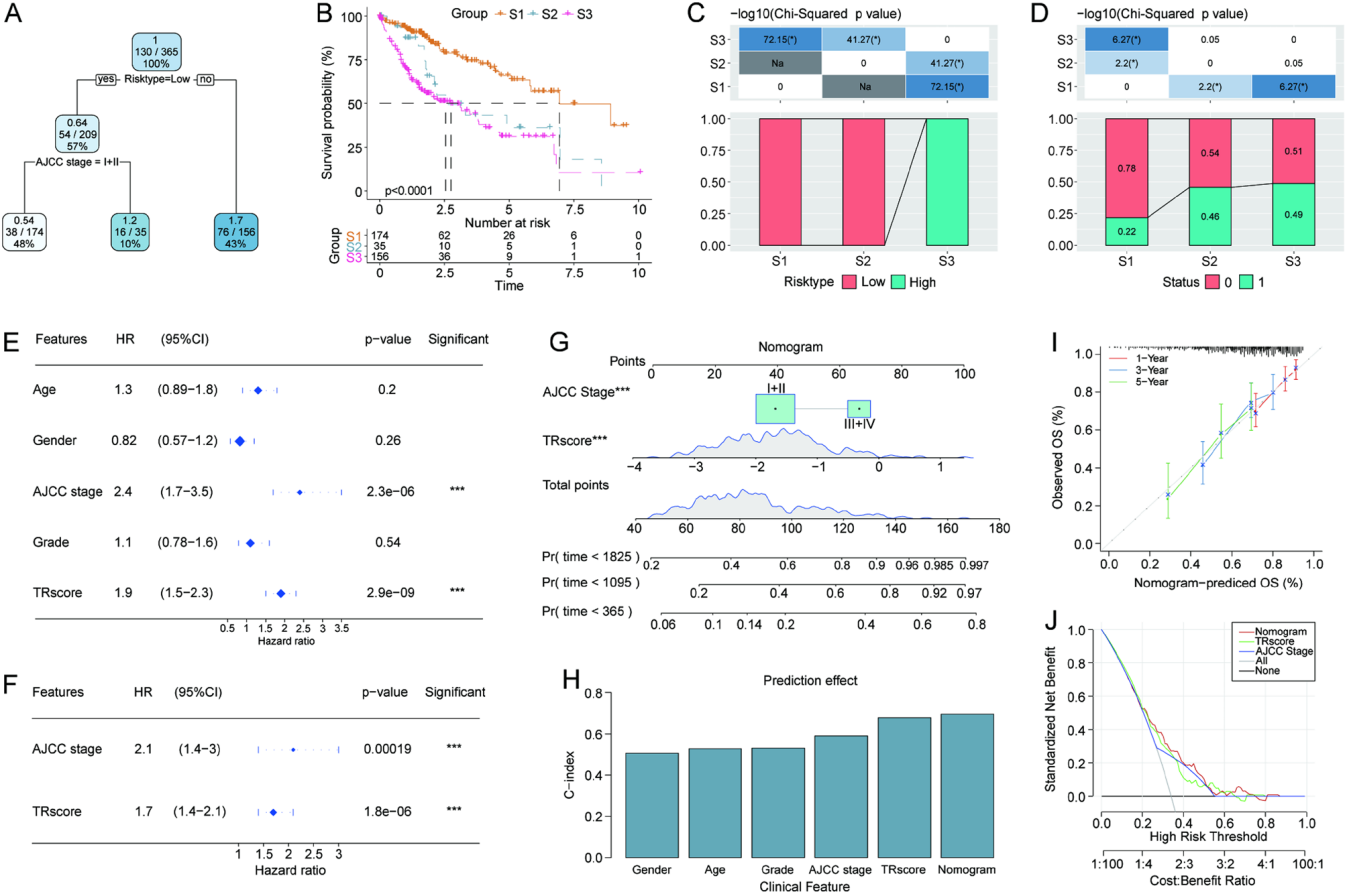
Improvement of the prognostic model. (**A**) Patients with full-scale annotations including risk type and AJCC stage were used to build a survival decision tree to optimize risk stratification. (**B**) Significant differences in overall survival were observed among the three subgroups. (**C**,**D**) Comparative analysis between the risk subgroups. (**E**) Univariate Cox regression analysis. (**F**) Multivariate Cox regression analysis. (**G**) Nomogram including AJCC stage and TRscore. (**H**) Comparison of concordance index. (**I**) Calibration curves and (**J**) the decision curve of the nomogram.

Using the TCGA cohort, a survival decision tree was constructed to optimize the risk model, by including five parameters: age, sex, AJCC stage, grade, and risk type. Ultimately, three subgroups (S1, S2, and S3) were generated using two parameters (AJCC stage and risk type) (Fig. 8A). The results revealed a significant difference in the overall survival rate among the three subgroups (Fig. 8B). The samples in the S3 subgroup had a high TRscore, while those in the S1 and S2 subgroups had low TRscores (Fig. 8C). In addition, the survival status of patients differed with risk subgroups (Fig. 8D). Univariate and multivariate Cox regression analyzes based on the TRscore and clinicopathological features showed that AJCC stage and risk type were identified independent risk factors for prognosis (Fig. 8E–F). Furthermore, to quantify the risk assessment and survival probability of patients with HCC, a nomogram was established using the two factors (Fig. 8G). The results demonstrated that the TRscore had the greatest impact on survival prediction. Based on the concordance index, the nomogram had better predictive performance (Fig. 8H). The calibration curve showed that the predicted values were close to the observed values in terms of 1-, 3-, and 5-year overall survival (Fig. 8I), indicating small prediction errors in the nomogram. Moreover, the decision curve showed that the benefit of the nomogram was significantly higher than that of the extreme curve, suggesting the strong clinical utility of the model in predicting the prognosis of patients with HCC (Fig. 8J).

### Validation and analysis of single-cell RNA sequencing (scRNA-seq) data

**Figure 9:**
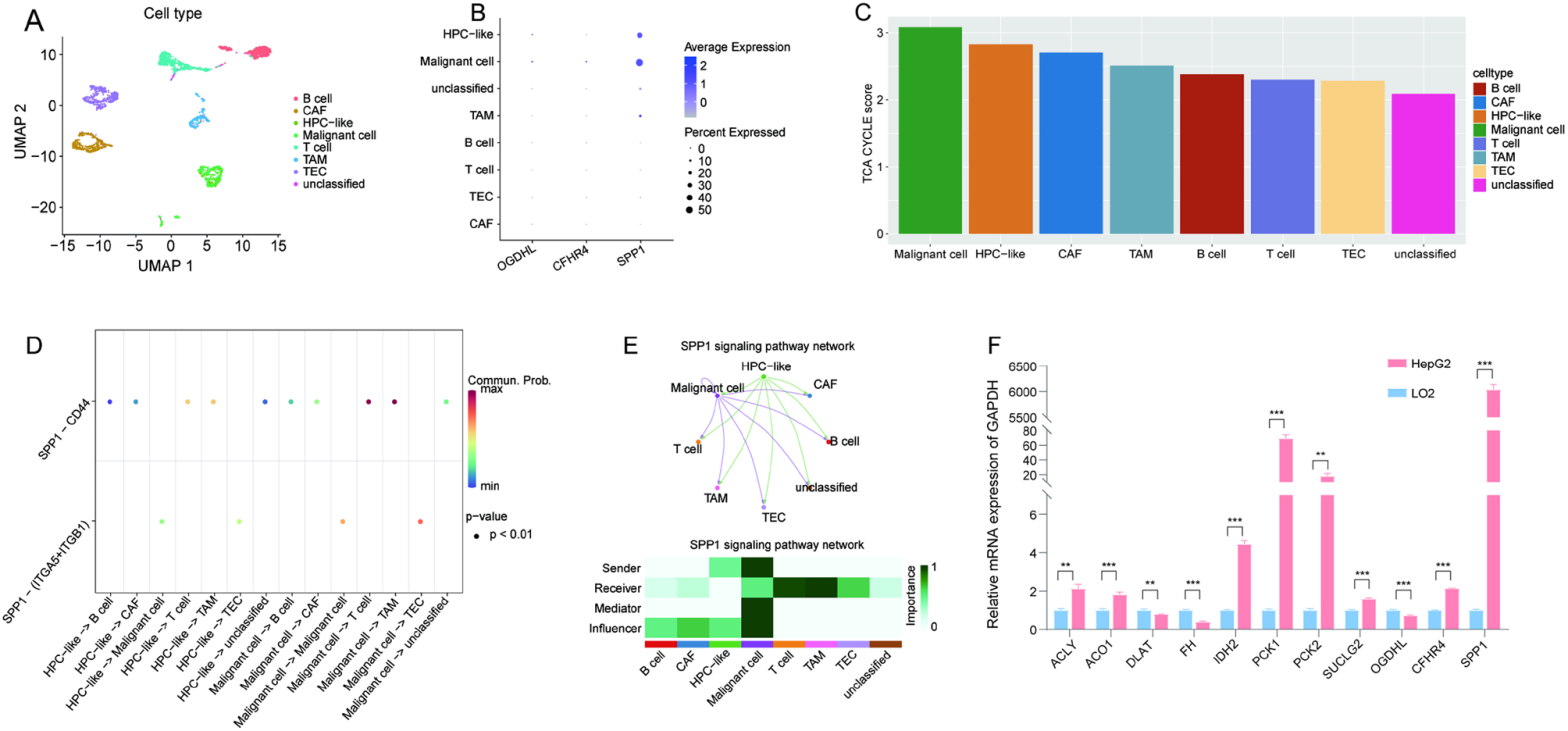
Validation and analysis of single-cell RNA sequencing data. (**A**) The UMAP plot displaying the proportion of cells in GSE125449 samples. (**B**) The expression of three genes identified using the TRscore model in cell subpopulations. (**C**) Differences in TCA cycle-related gene enrichment score among cell subpopulations. (**D**,**E**) Intercellular communication. (**F**) Expression of prognostic genes in TCA cycle and signature genes identified using TRscore.

To further study the robustness of the risk model and explore immune infiltrating cells, scRNA-seq data from the GSE125449 dataset were analyzed. Based on the cell annotation data, cells were divided into eight types of cell subpopulations (Fig. 9A)^24^. Then, the expression of three genes was mapped using the TRscore model in different cell subpopulations. The results revealed that *SPP1* was highly expressed in malignant cells and hepatic progenitor cell (HPC)-like cells (Fig. 9B). Using the ssGSEA method, the TCA cycle-related gene enrichment score was calculated for each cell subpopulation, and the results showed that the malignant cells had the highest score (Fig. 9C). CellChat was used for cell communication analysis to explore the signaling pathway network between neoplastic cells and immunocytes. The findings demonstrated that there were more connections among malignant cells, HPC-like cells, and immunocytes (Fig. S4). In particular, ligand-receptor pair analysis revealed that the SPP1 signal was mainly involved in the interaction among tumor-associated macrophage (TAM), T cells, and malignant cells via SPP1–CD44 and SPP1–(ITGA5 + ITGB1) ligand-receptor pairs (Fig. 9D–E).

### In vitro experimental validation of TCA cycle-related genes and three signature genes

The expression of nine genes associated with prognosis in the TCA cycle and signature genes of TRscore was analyzed using hepatoma cells (HepG2) and normal hepatocytes (LO2) for quantitative real-time polymerase chain reaction (qRT-PCR) analysis. The mRNA levels of these 11 genes (*OGDHL* was also used to construct the TRscore) differed significantly between hepatocytes and hepatoma cells (Fig. 9F). Consistent with our previous findings, *ACLY* and *SPP1* expression was significantly higher in HepG2 than in LO2, whereas *OGDHL* and *FH* expression was higher in LO2. These findings suggest the potential utility of these genes as biomarker candidates or potential therapeutic targets for patients with HCC. The experimental results at the cellular level are for reference only. In general, the verification results support our model.

## Discussion

HCC is characterized by a high recurrence rate and mortality, owing to which it is associated with a substantial burden worldwide. To date, choosing a reasonable and an effective treatment approach is an important challenge in the clinical decision-making of patients with HCC. Therefore, it is of great significance to seek efficient prediction methods for evaluating the prognosis and guiding the treatment of patients. In recent years, metabolic reprogramming has been recognized as a common feature of several tumors. Moreover, the TCA cycle has been found to be a common pathway for the metabolism of the three major nutrients and is also a hub for the synthesis and transformation of carbohydrates, lipids, and amino acids. Accordingly, the identification of genes related to the TCA cycle may provide valuable insights into the molecular mechanism, prognostic evaluation, and clinical management of HCC.

Accordingly, the present study first identified TCA cycle-related genes that are significantly associated with prognosis using data from public databases and obtained three molecular subtypes of HCC via clustering analysis. Among the nine genes associated with prognosis, *OGDHL* was found to be highly expressed in tumors with good differentiation or smaller size. Reportedly, mitochondrial dysfunction and subsequent metabolic disorders are common in HCC and result from mutation or silencing of genes encoding key enzymes in the TCA cycle. Following the inhibition of oxidative metabolism of glucose and glutamine, the primary carbon source for cancer cell growth shifts to reductive glutamine metabolism. Accordingly, Weiqi et al. have revealed that silencing *OGDHL* can induce the metabolism of reductive glutamine by inhibiting the activity of mitochondrial multi-enzyme OGDH complex, thus contributing to the development and progression of HCC^19^. On the contrary, *ACLY*, as a potential risk gene for prognosis, is overexpressed in various cancers such as HCC, lung cancer, bladder cancer, prostate cancer, colorectal cancer, and gastric cancer^25,26^. As the most important enzyme linking glucose catabolism with cholesterol and fatty acid anabolism, *ACLY* plays a crucial role in metabolic reprogramming by depriving cytosolic citrate to enhance glycolysis^27^. Reportedly, *ACLY* is highly expressed in liver tumor-initiating cells and promotes HCC metastasis by regulating the Wnt/β-catenin axis^28^. Moreover, *ACLY* inhibitors in combination with other chemotherapeutic drugs have shown anti-proliferative effects in vitro^29^. However, further trials are still required to evaluate the role of *ACLY* inhibitors in the treatment of HCC. Thus, further studies on these prognostic genes may provide clues for the development of new therapies.

Next, we evaluated DEGs between the three molecular subtypes and identified three genes (*OGDHL*, *CFHR4*, and *SPP1*) for constructing the prognostic TRscore model by screening and compression. The model showed excellent predictive performance in different datasets and may help guide the prognostic evaluation and predict pathological features of HCC. In addition, the TRscore showed a wide range of universality in 32 cancer types. Reportedly, several studies have proposed the latent effects of these genes in HCC. For example, secreted phosphoprotein 1 (*SPP1*), also known as osteopontin, is an extracellular matrix protein that is closely related to tumor invasion and metastasis, apoptosis inhibition, and angiogenesis. A recent study has suggested that the hypoxic microenvironment upregulates *SPP1* expression, which results in cancer-associated fibroblasts and *SPP1* macrophages forming a tumor immune barrier. The interaction between the two stimulates extracellular matrix remodeling and affects the efficacy of immune checkpoint blockade by limiting the infiltration of immunocytes into malignant areas^30^. This indicates that the signatures that constitute the TRscore may help evaluate the TME and immunotherapy effect.

Based on the TRscore, the patients were divided into high- and low-risk groups. Enrichment analysis demonstrated that pathways related to cell cycle, immune inflammation, and tumor development were more upregulated in the high-risk group, whereas metabolic pathways including xenobiotic metabolism, fatty acid metabolism, bile acid metabolism, and adipogenesis were significantly enriched in the low-risk group. An analysis of somatic mutations showed that the mutations of *TP53* and dedicator of cytokinesis protein 2 (*DOCK2*), which are associated with detrimental biological behavior, were more abundant in the high-risk group than in the low-risk group. Specifically, TP53 is a star gene that regulates the cell cycle and induces apoptosis. Shuai et al. have found that DOCK2 knockdown leads to the depletion of CD8^+^T-cell in tumors, which can be reversed by tolazamide, thus enhancing the efficacy of anti-PD-L1 antibody plus apatinib in the treatment of HCC^31^. These findings help identify important regulators and potential therapeutic targets in the immunotherapy of HCC. The rational use of existing or underdevelopment therapies may be an important aspect of enhancing the anti-tumor response; therefore, we conducted an in-depth exploration of treatment strategies. The combination of TMB and TRscore demonstrated that H-TMB + H-risk often indicated a worse outcome. However, ssGSEA analysis suggested that the high-risk group was associated with higher levels of immune infiltration, immune checkpoint expression, and anti-cancer immunotherapy activity. This suggests that patients with TRscores may respond more positively to immune checkpoint therapy. Besides, sensitivity analysis of traditional chemotherapies has revealed that the high-risk group was sensitive to midostaurin, saracatinib, bortezomib, and dasatinib, whereas the low-risk group was sensitive to erlotinib and phenformin. These findings indicate the potential application of the TRscore model in selecting personalized treatments for patients with HCC.

The analysis of scRNA-seq data showed that SPP1 was highly expressed in malignant cells and HPC-like cells and mainly involved in the interaction among tumor-associated macrophage, T cells, and malignant cells via SPP1–CD44 and SPP1–(ITGA5 + ITGB1). This finding provides ideas and strategies for further exploring the interaction network within TME.

In summary, using TCA cycle-derived subtypes, we identified DEGs among molecular subtypes to establish a new gene signature to stratify HCC patients. The developed model showed outstanding ability for evaluating prognostic outcomes, immunosuppressive status, and treatment response. However, our study was based on public databases and has a few limitations. We only verified the expression of partial genes at the cellular level. Further research is needed to explore the potential relationship between TRscore and HCC prognosis as well as the underlying mechanism. In conclusion, the novel model provides an alternative strategy for clinical decision-making and risk stratification in patients with HCC.

## Conclusion

In conclusion, we identified three subtypes of HCC using the TCA cycle-related genes that demonstrated significant prognostic ability as well as established a risk model based on DEGs. TRscore revealed the differences in survival probability, clinicopathology, genomic characteristics, TME, functional pathways, immunotherapy response, and drug sensitivity between high- and low-risk patients. The findings indicate that TRscore might be a useful tool to assist in individualized treatment and clinical management of patients with HCC.

## Methods

### Data collection

The latest gene expression data and the accompanying clinical follow-up information of the TCGA-LIHC dataset (n = 365) were downloaded from TCGA database. The dataset contains information on *FPKM* expression profiles obtained from RNA-Seq samples, as well as mutect2-treated stable nuclear variant mutations. For validation datasets, the raw survival data obtained from HCCDB18 (n = 203) and GSE14520 (n = 232) datasets were used and were extracted from Hepatocellular Carcinoma and Gene Expression Omnibus (GEO) databases, respectively^32^. Data on TCA cycle-related pathways and corresponding 31 genes (KEGG_CITRATE_CYCLE_TCA_CYCLE) were obtained from the GSEA database (Table S1)^33^. Data on single-cell sequencing were extracted from the GSE125449 dataset (n = 9).

Samples with incomplete survival status and follow-up data were excluded from the analysis.

### Consensus clustering analysis

The consistency matrix was constructed by consensus clustering analysis using the ConsensusClusterPlus package. Samples were clustered and typed, with the parameters being clusterAlg = “km” and distance = “euclidean”. The sampling was repeated 500 times, with a sampling ratio of 80% per repeat.

The samples were clustered using the consensus clustering analysis, and the optimal number of clusters was determined on the basis of the CDF. Analysis of the CDF delta area curve enabled the selection of relatively stable results.

### Construction of prognostic models

DEGs were analyzed using the “limma” R package, with |log2FC|> 1 and statistical significance at *p* < 0.05 (Fig. 5A). To reduce the gene count of the risk models, the Lasso-Cox algorithm was used to further compress the selected genes using the “glmnet” R package, followed by a tenfold cross-validation. Subsequently, multiple stepwise regression analysis was used to shrink gene count to identify the key genes that affect prognosis.

The following formula was used to calculate the TRscore for each patient:

$${\text{TRscore}} = \Sigma \beta {\text{i}} \times {\text{Expi}}$$

where β is the Cox regression coefficient of the corresponding gene.

Functional enrichment analysis was performed using GSEA (h.all.v2023.1.Hs.symbols.gmt) and Kyoto Encyclopedia of Genes and Genomes databases.

### ROC curve and the optimal cut-off value

The TRscore of each sample was calculated based on its expression level, and the optimal cut-off was determined using the “surv_cutpoint” survminer package, which helps classify high- and low-risk groups. Then, the “timeROC” R package was used to perform ROC analysis. Furthermore, the ROC analysis was used for prognostic categorization.

### Mutation information

Gene mutation datasets were downloaded from the TCGA database. Briefly, the “mafCompare” maftools package was used to compare differentially expressed mutated genes from two different cohorts, which were analyzed using Fisher’s test to calculate TMB^34^. Patients with different TMBs were compared to explore the impact of TMB on patients’ overall survival. Furthermore, the relativity of TMB and TRscore was analyzed to investigate the combined effect of the two parameters on prognosis.

### Prediction of immunotherapy response and chemotherapeutic susceptibility

ssGSEA was performed using the “GSVA” R package to investigate the immune function^33^. In the present study, we obtained 28 and 27 immune-related gene sets and their corresponding markers from the previous two studies^35,36^. The enrichment score obtained using ssGSEA indicates the relative abundance of each immunocyte and anticipates the impact of immune cells on prognosis. Accordingly, ssGSEA was used to determine the response to immunotherapy, chemotherapy, and targeted therapy, as well as to calculate the enrichment score of genes related to the TCA cycle.

Besides, TIMER algorithm was used to evaluate immune infiltration between subtypes. The TIDE score was determined to assess the possibility of HCC cells escaping the immune system^37^. For this, the TIDE assessment was conducted to predict the effect of immunotherapy on the high- and low-risk groups.

The susceptibility of HCC cells to various chemotherapeutic drugs in different risk groups was evaluated using the “pRRophetic” R package to determine the half-maximal inhibitory concentration, using the Wilcoxon signed-rank test^38^.

### Optimization of risk model

To classify the subgroups according to clinicopathologic characteristics and TRscore, a decision tree was constructed using the “rpart” R package. In addition, a nomogram was constructed using the “rms” R package and integrated risk factors and independent prognostic factors for survival prediction. The calibration and decision curves were plotted using the “timeROC” and “rmda” R packages to confirm the diagnostic value and clinical utility of the nomogram.

### scRNA-seq data treatment and analysis

scRNA-seq data were filtered to calculate the percentage of mitochondria and rRNA using PercentageFeatureSet; this ensured that each gene was expressed in at least three cells and each cell between 200 and 3000. The proportion of mitochondria in each cell should be ≤ 10% and the unique molecular identifier of each cell should be ≥ 200. The data were normalized and highly variable genes were shortlisted using log-normalization and FindVariableFeatures functions, respectively. Genes were scaled using ScaleData, and anchor points were identified via dimension reduction using PCA, with the dim value set to 20. Then, the batch correction was executed using Harmony. The average gene expression level of each cell subpopulation was calculated using the AverageExpression. CellChat was used for inference and analysis of cell–cell communication^39^.

### Cell culture

The hepatoma cell line HepG2 and normal hepatocyte line LO2 were procured from the Chinese Academy of Sciences (Shanghai, China). The cells were cultured separately in Dulbecco’s modified Eagle’s medium (Gibco, USA) and RPMI-1640 medium (Gibco, USA) with 10% fetal bovine serum (Gibco, USA) and 1% penicillin/streptomycin (Beyotime, China) at 37 °C in a 5% CO_2_ incubator.

### The process of qRT-PCR

The total RNA was extracted from cells using the RNA Easy Mini Kit (QIAGEN, China), based on the manufacturer’s instructions. The obtained total RNA was then reverse-transcribed into cDNA using the PrimeScript RT Master Kit (Takara, Japan). The DNA was amplified using the TB Green Premix (Takara, Japan), and relative mRNA expression levels were calculated using the 2 − ΔΔCT method, with *GAPDH* as the internal reference. The sequences of primers used are presented in Table S2.

### Statistical analysis

Bioinformatics analyses were conducted using the R software (version 3.6.0). The two-group difference was tested using the Wilcoxon signed rank test or student's t test. The Kruskal–Wallis test was used to calculate differences among the three groups. Categorical variables were compared using the χ^2^ test. Differences in survival time among groups were compared using the log-rank test. Results with a *p*-value of < 0.05 were considered statistically significant.

### Supplementary Information

Supplementary Information.

## Data Availability

All data involved in this study are available from the corresponding author on request. The RNA sequencing data were obtained from the TCGA database (https://www.cancer.gov/tcga), GEO database (GSE14520 and GSE125449, https://www.ncbi.nlm.nih.gov/geo/), and HCCDB database (HCCDB18, http://lifeome.net/database/hccdb/).
