# Supplementary material for: Prognostic and immune predictive roles of a novel tricarboxylic acid cycle-based model in hepatocellular carcinoma
Source: Sci Rep. 2024 Jan 28;14:2333. doi: 10.1038/s41598-024-52632-0 (PMC10822853; doi:10.1038/s41598-024-52632-0)
Supplement: Supplementary file 1 — Supplementary Information. [file 41598_2024_52632_MOESM1_ESM.docx]

Supplementary material

**Prognostic and immune** **predictive roles of a novel tricarboxylic acid cycle-based model in hepatocellular carcinoma**

Yifan Zeng^1#^, Tao Yu^1#^, Shuwen Jiang^1^, Jinzhi Wang^1^, Lin Chen^1^, Zhuoqi Lou^1^, Liya Pan^1^, Yongtao Zhang^1^, Bing Ruan^1^*

^1^ State Key Laboratory for Diagnosis and Treatment of Infectious Diseases, National Clinical Research Center for Infectious Diseases, National Medical Center for Infectious Diseases, Collaborative Innovation Center for Diagnosis and Treatment of Infectious Diseases, The First Affiliated Hospital, Zhejiang University School of Medicine, Hangzhou City 310003, China

^#^These authors contributed equally.

***Corresponding author:** Bing Ruan

State Key Laboratory for the Diagnosis and Treatment of Infectious Diseases, The First Affiliated Hospital, Zhejiang University School of Medicine, 79 Qingchun Rd., Hangzhou City 310003, China. Tel and Fax: +8657187236585

E-mail: ruanbing@zju.edu.cn.

**Supplementary figures**

**
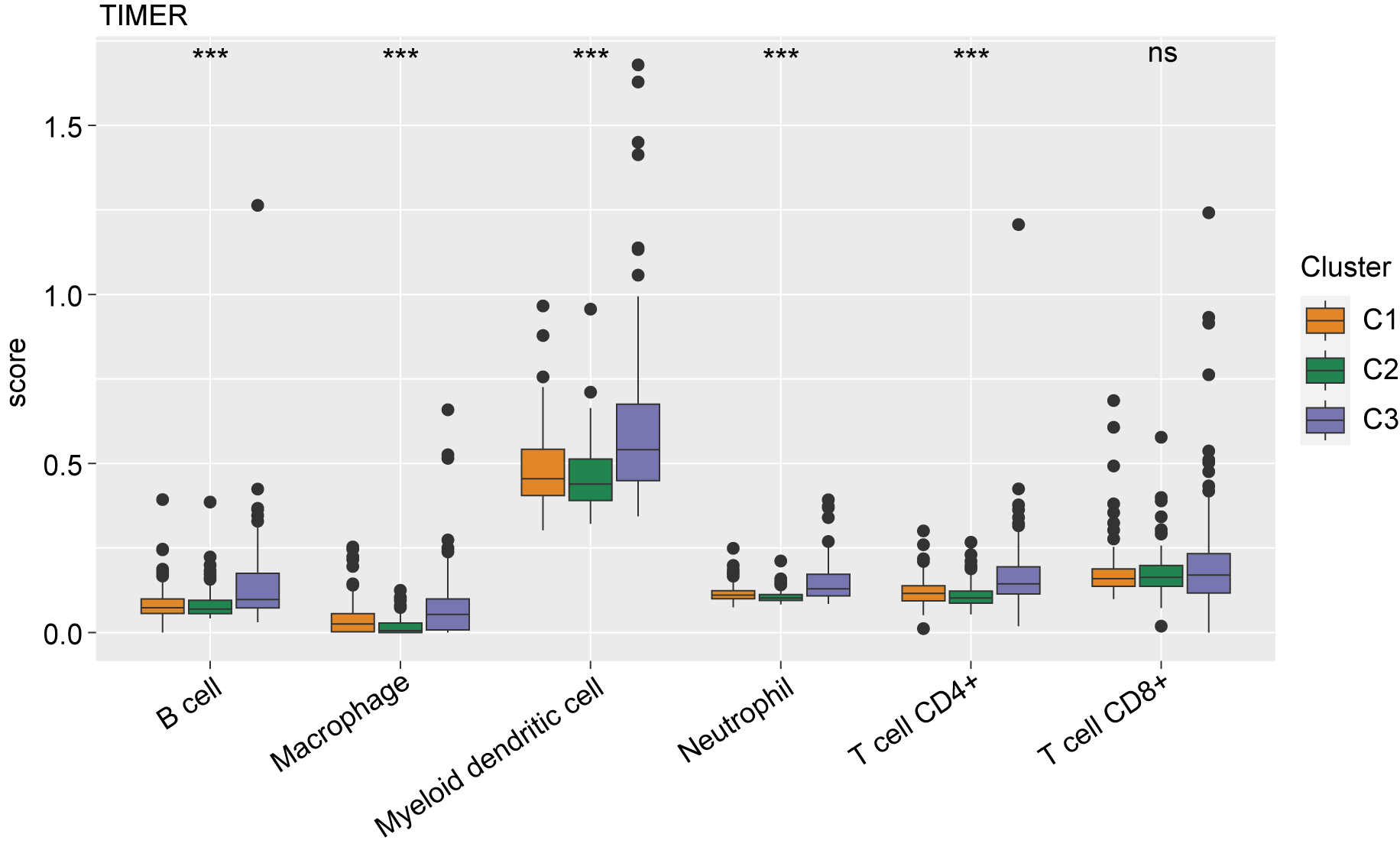
**

**Figure S1.** Immune infiltration analysis based on Tumor Immune Estimation Resource (TIMER) algorithm.


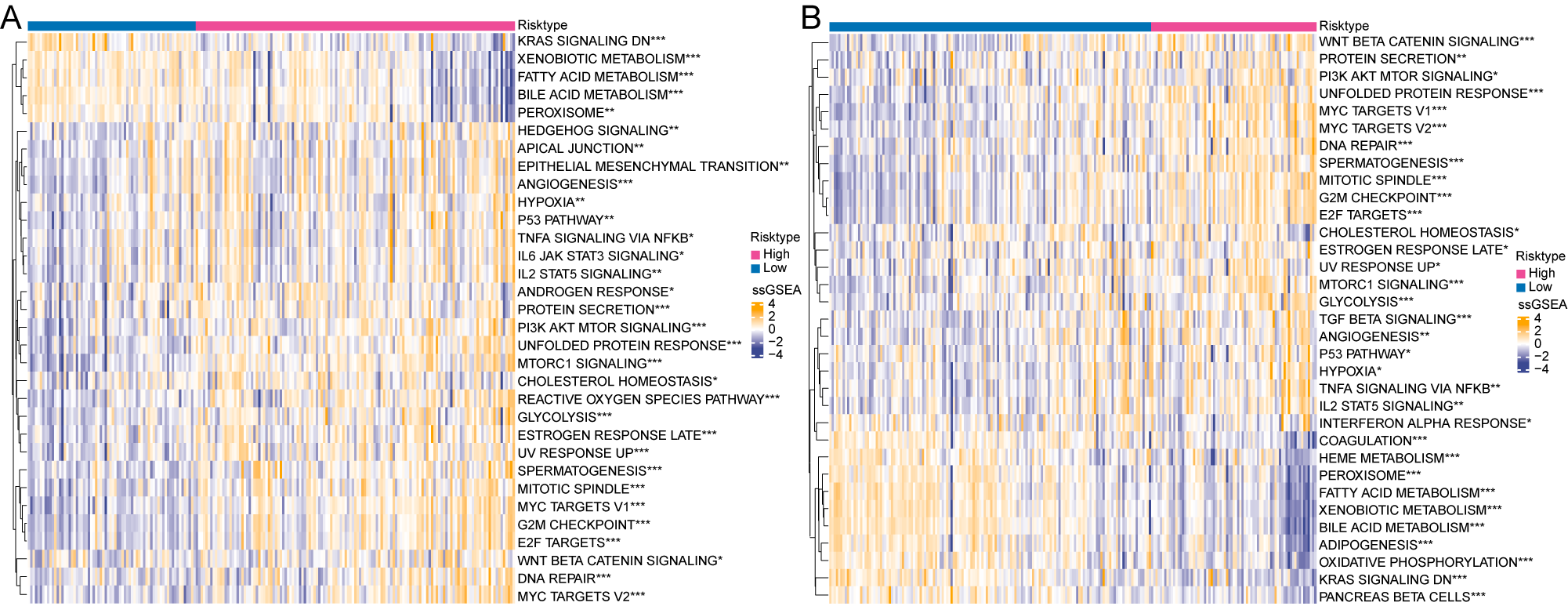


**Figure S2.** Biological functions enriched in different risk subgroups based on the HCCDB18 (A) and GSE14520 (B) datasets.


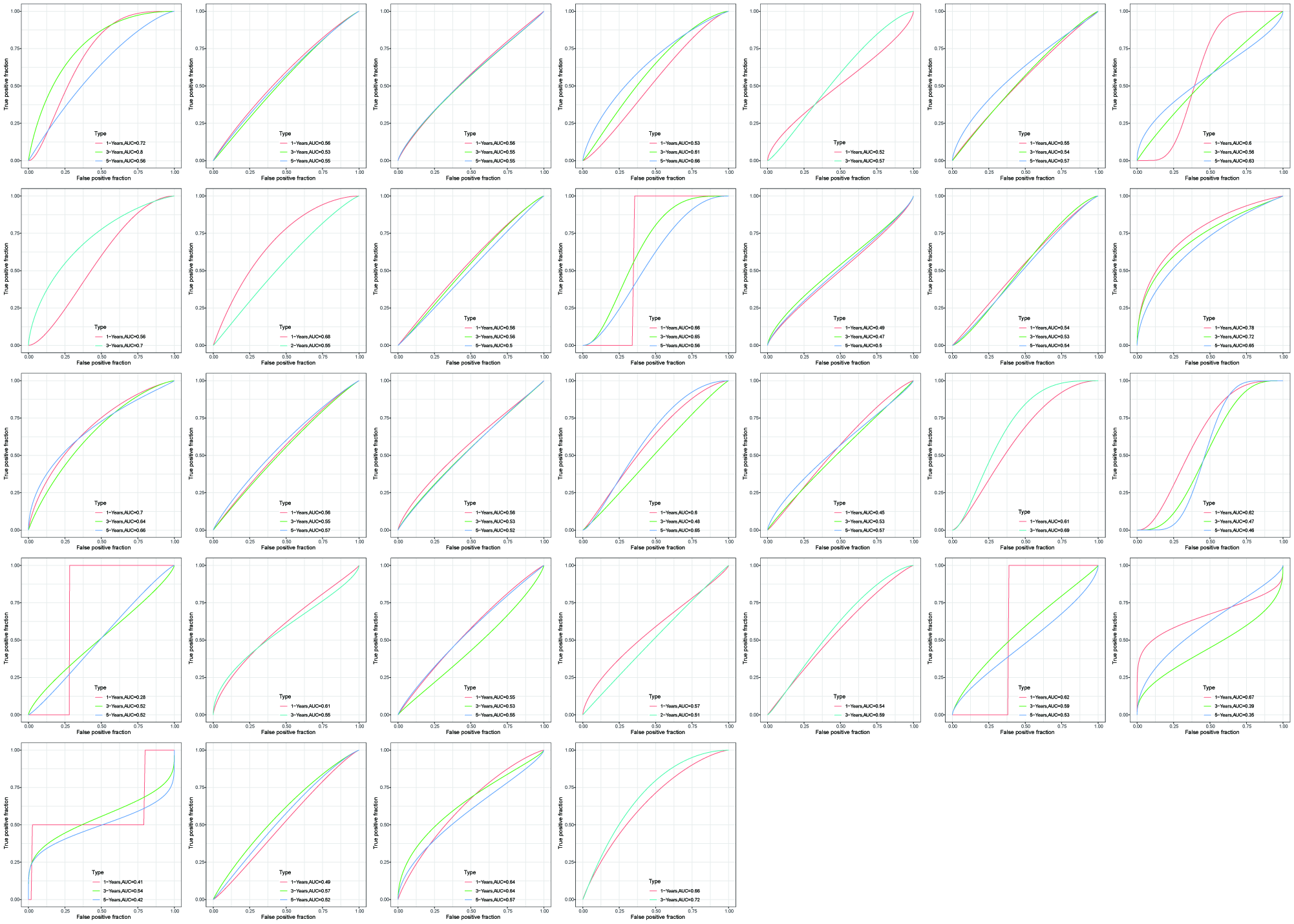


**Figure S3.** ROC curves of each cancer type in predicting patient outcome across cancer types.


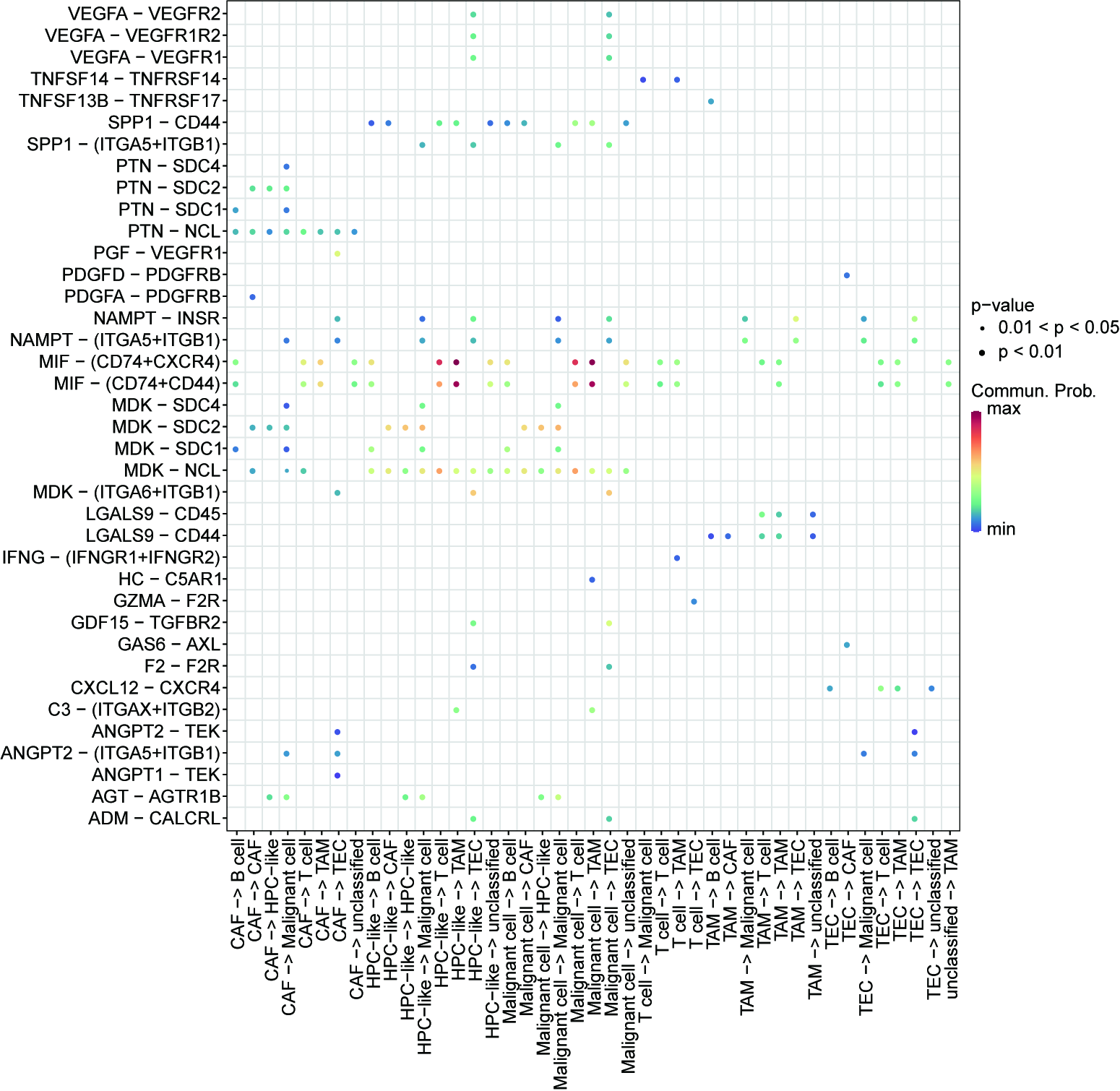


**Figure S4.** Intercellular communication among different cell subpopulations.

**Supplementary Table1.** The gene set of 31 tricarboxylic acid cycle-related genes

| No. | Gene | No. | Gene |
| --- | --- | --- | --- |
| 1 | ACLY | 17 | OGDHL |
| 2 | ACO1 | 18 | PC |
| 3 | ACO2 | 19 | PCK1 |
| 4 | CS | 20 | PCK2 |
| 5 | DLAT | 21 | PDHA1 |
| 6 | DLD | 22 | PDHA2 |
| 7 | DLST | 23 | PDHB |
| 8 | FH | 24 | SDHA |
| 9 | IDH1 | 25 | SDHB |
| 10 | IDH2 | 26 | SDHC |
| 11 | IDH3A | 27 | SDHD |
| 12 | IDH3B | 28 | SUCLA2 |
| 13 | IDH3G | 29 | SUCLG1 |
| 14 | MDH1 | 30 | SUCLG2 |
| 15 | MDH2 | 31 | SUCLG2P2 |
| 16 | OGDH |  |  |

Note: The gene set was downloaded from the following site: https://www.gsea-msigdb.org/gsea/msigdb/human/geneset/KEGG_CITRATE_CYCLE_TCA_CYCLE.

**Supplementary Table2.** The information of primers sequences for qRT-PCR.

| Primer name | Sequence (5'-3') | Base |
| --- | --- | --- |
| DLAT-F | GCGACGGGCTCAGAATGTA | 19 |
| DLAT-R | GTCACGCTGTTGCGACGAG | 19 |
| ACLY-F | CACCGAAGACCAACATCCACA | 21 |
| ACLY-R | AAATACAGCAGGTAGCAGAGCAAA | 24 |
| PCK1-F | GGGTGGAAGGTTGAGTGCG | 19 |
| PCK1-R | CCAGGAGCGACACCGAAA | 18 |
| OGDHL-F | CATCACTCTGTCGCTGGTTGC | 21 |
| OGDHL-R | CGCTCAGGTGGAAGGTCTCAT | 21 |
| PCK2-F | AGAGGTGTTGGCTGAGCTTGA | 21 |
| PCK2-R | GTCTATGGCACATTGAAGATGTTAGT | 26 |
| FH-F | TCCAGCCGCAGCCTTAGC | 18 |
| FH-R | TCATTTGGCACCTTTAGTTCACC | 23 |
| IDH2-F | CGTATTATCTGGCAGTTCATCAAGG | 25 |
| IDH2-R | GGTCATCAGTCTGGTCACGGTTT | 23 |
| ACO1-F | AGTAATCCGTCTGTGATGTTAGGG | 24 |
| ACO1-R | CATGACTCCGCTTTCTTGTAGGT | 23 |
| SUCLG2-F | GGAGGTGGTGTAAAGGAAGCTC | 22 |
| SUCLG2-R | TGATGGCACAGTTGACGATACC | 22 |
| CFHR4-F | GGGGATACCATTGAATTTATGTGTA | 25 |
| CFHR4-R | TCCACTATGCCTTCCCTACACA | 22 |
| SPP1-F | GTGCATACAAGGCCATCCC | 19 |
| SPP1-R | CCTGACTATCAATCACATCGGAAT | 24 |
| GAPDH-F | CGCTGAGTACGTCGTGGAGT | 20 |
| GAPDH-R | TGCTGATGATCTTGAGGCTGTTG | 23 |
